# Psychological Burden and Experiences Following Exposure to COVID-19: A Qualitative and Quantitative Study of Chinese Medical Student Volunteers

**DOI:** 10.3390/ijerph18084089

**Published:** 2021-04-13

**Authors:** Kaiting Zhang, Yixiang Peng, Xiaowei Zhang, Liping Li

**Affiliations:** 1 Injury Prevention Research Center, Shantou University Medical College, Shantou 515041, China; 19ktzhang@stu.edu.cn (K.Z.); 19yxpeng@stu.edu.cn (Y.P.); 20xwzhang1@stu.edu.cn (X.Z.); 2 School of Public Health, Shantou University, Shantou 515041, China

**Keywords:** COVID-19, medical student volunteers, qualitative research, psychological burden

## Abstract

*Background*: During the COVID-19 pandemic, some medical students devoted themselves to volunteer activities, but it was the first time that they had been exposed to such an infectious disease and they might have experienced fear in the face of the epidemic. We aimed to conduct a timely assessment of the psychological burden and experience on medical student volunteers during the COVID-19 pandemic. *Methods*: We used the 21-item Depression Anxiety Stress Scales to survey the psychology burden of students in April 2020. Semi-structured interviews were conducted with nine medical students who signed up for volunteer activities in Chinese from February to April 2020. Quantitative and qualitative methods were used to analyze the data. *Results*: The detection of depression, anxiety and stress of medical student volunteers were 26.8%, 20.2% and 11.1%, respectively. The volunteer’s negative emotions were more pronounced before work and diminished gradually. Most participants expressed no concern about being infected themselves, but worry about family infection. Participant’s motivations for volunteering were primarily their duties as medical students and encouragement from their families/teachers. The vast majority of medical students said they would be willing to work as medical assistants again and this experience would not affect their career choice. *Conclusions*: Chinese medical student volunteers tended to show negative emotions at the beginning of their work, and then gradually declined, while positive emotions emerged. Most medical students were willing to volunteer as medical assistants when their country needed them due to their sense of responsibility as medical students. This study on the psychological and experiential aspects were derived from Chinese medical student volunteers and might have a significant impact on future public health emergencies in similar settings.

## 1. Introduction

Since the pandemic triggered by coronavirus disease 2019 (COVID-19), governments have taken increasing action to slow the spread of the infection, to save as many lives as possible, and to buy crucial time for the development of potential treatments, healthcare capacity and/or vaccine production by making information about the outbreak public [1,2,3]. The COVID-19 outbreak had created considerable panic, and all the evidences indicated that this disease was more dangerous than severe acute respiratory syndrome (SARS) in 2003 [4]. During this period, medical workers were not only at risk of infection to themselves and their families, but also under increasing workload pressure [5,6]. There came a point at which medical needs outpaced the capacity of medical staff, and non-professionals had to be included in the healthcare workforce as well. Not only did military or retired medical professionals need to be recruited for healthcare, but others, such as medical students, were also enlisted [1].

The COVID-19 pandemic brought into focus the mental health of various affected populations [7,8]. Although several studies have assessed mental health issues during COVID-19 epidemics, most have focused on health workers, patients, children, and the general population [9,10,11]. For example, a study by Dong et al. showed that about a quarter of (24.2%) medical staff experienced psychological problems during the COVID-19 pandemic [4]. Guo et al. [12] found that the levels of anxiety and depression among Chinese patients during the early stage of the COVID-19 pandemic were 55.3% and 60.2%, respectively. Wang et al. [13] investigated the general population at the beginning of the epidemic and four weeks after the peak of the outbreak, and found that the incidence of moderate-to-severe stress, anxiety and depression was noted in 8.1%, 28.8% and 16.5% of the population, respectively, in the initial evaluation, while the longitudinal changes in stress, anxiety and depression levels were not significant. These studies suggest that there was an upward trend of psychological burden during the outbreak period.

During the COVID-19 epidemic, medical personnel were under unprecedented pressure. With the development and severity of the epidemic, some medical students used their professional knowledge to devote themselves to voluntary epidemic prevention and control activities. As future medical workers, they bore the responsibility and obligation to do so, with the hope that they would be able to serve the public with what they had learned and develop their practice during the pandemic, leading to self-improvement. However, at the same time, as the younger generation, it was the first time that they had been exposed to such a large infectious disease and, like the general public, they were fearful in the face of the epidemic [14]. Therefore their psychology of volunteering during COVID-19 and their experiences deserved our study and attention. Additionally, at the time, the idea of involving medical students as medical assistants in the prevention and control of sudden infectious diseases was still controversial. Thus far, no study has yet focused on analyzing the psychological conditions of medical students during the COVID-19 remission period, including conducting in-depth interviews with medical student volunteers.

Therefore, the purpose of this study was to understand the psychological impact on this particular group of medical student volunteers during the epidemic and to analyze their experiences during this period using the framework analysis method to deeply understand the meaning and essence of volunteer’s experiences. This paper documented the results of questionnaires and interviews carried out with medical student volunteers from a medical college in China, both qualitatively and quantitatively. Through recognizing the factors affecting medical student volunteer’s mental health, this study provides recommendations regarding whether medical student volunteers should be included in future epidemic prevention and control efforts and will be a basic resource that provides a scientific basis for targeted psychological interventions for medical student volunteers in the hope that safer workplaces can be established in order to protect medical student volunteers.

## 2. Materials and Methods

### 2.1. Participants

This was a quantitative and qualitative study, involving semi-structured and in-depth interviews with medical students in China. Firstly, we conducted a survey of undergraduate and graduate students in a medical college from 16 to 26 April. Students were invited to fill out the questionnaire via WeChat. Next, we screened out students who indicated that they had actively participated in a prevention and control assignment during the epidemic (including disseminating knowledge about epidemic prevention to others, assisting relevant institutions to conduct epidemiological investigations, taking people’s temperatures, etc.). A total of 1041 subjects who had been actively involved in the epidemic prevention and control were included in our study.

Secondly, nine medical student volunteers who assisted in relevant departments and were potentially exposed to COVID-19 during the epidemic were selected purposefully by the researchers. Interviews with participants were conducted in Chinese from 1 October to 15 November 2020. The selected participants were interviewed after the first author explained the study objectives and obtained their verbal consent. Participants were asked about their psychological feelings and experiences as volunteers during the outbreak (two separate questions).

### 2.2. Instruments

#### 2.2.1. The Quantitative Study Used 21-Item Depression Anxiety Stress Scales (DASS-21)

The Chinese versions of DASS-21 was used to assess participant’s negative emotion in the quantitative study. The purpose of the questions was to “assess the severity of the core symptoms of depression, anxiety, and stress” [15]. Sum scores were computed by adding up the scores on the items per (sub)scale and multiplying them by a factor of 2. Sum scores for the total DASS-21 thus range between 0 and 120, and those for each of the subscales may range between 0 and 42 [16]. Answers were reported on a four-point Likert scale (0–3). A score of 0 indicated that the item “did not apply to them”, and a score of 3 meant that the participant considered the question to apply “very much, or most of the time” [1]. For depression, the criteria were normal (0–9 points), mild (10–13 points), moderate (14–20 points), severe (21–27 points), and extremely severe (28+ points). For anxiety, the standards were normal (0–7 points), mild (8–9 points), moderate (10–14 points), severe (15–19 points), and extremely severe (20+ points). For stress, the criteria were normal (0–14 points), mild (15–18 points), moderate (19–25 points), severe (26–33 points), and extremely severe (34+ points) [17]. Cronbach’s alpha coefficients for 3 sub-scales were 0.915 (depression), 0.920 (anxiety), and 0.915 (stress) in this study.

#### 2.2.2. The Qualitative Study

The participants were asked some key questions, such as “Please describe the psychological feelings of exposure to suspected/confirmed cases”, and “Please talk about whether the epidemic will affect your choice of employment direction in the future”. The interview continued according to the answers provided by the participant, and, on the basis of these answers, more in-depth questions such as “what do you mean?”, and “please explain in detail” were asked [6].

### 2.3. Procedures

The interviews were conducted by two postgraduate researchers trained in qualitative methods. All interviews were conducted via face-to-face or telephone interviews and were audio recorded. All participants provided verbal informed consent before the interviews began and were not compensated for their participation. Sampling and data collection continued until data saturation was reached, at which point no new data were obtained from the interviews [6]. Data saturation is measured from both the respondent’s and the researcher’s perspectives. On the one hand, it refers to the saturation of the respondent’s point of view, if the interviewee has finished talking or has nothing relevant to express, and on the other hand, if the researcher believes that the information obtained has reached their understanding of the problem or is sufficient to analyze their research objectives, then it is information saturation [18]. This study was approved by the Ethics Committee of Shantou University Medical College (project no. SUMC-2020-01).

### 2.4. Data Collection and Analysis

Descriptive statistics were compiled to describe participant’s demographics (e.g., age, gender, and academic year) and pie charts were used to describe the distribution of negative emotions among the participants.

All interviews were audio recorded with participant consent and lasted approximately 40 min. A teacher (LL) and two graduate students (KZ and YP) collected the data after receiving data collection training. Qualitative recordings were transcribed verbatim and analyzed using the seven steps of the framework analysis method i.e., transcription (converting recording to text version); familiarization (familiarizing ourselves with the entire interview process by recording, transcribing material and reflective notes); coding (coding all relevant things from as many different perspectives as possible); developing a framework (coding the first few records separately to form a set of codes that could be applied to all subsequent records and keeping an “other” code under each category to avoid ignoring inappropriate data); applying the framework (using the existing categories and codes for analytical application); charting and then interpreting data (coding the interview data and analyzing the links between categories to explore relationships and/or causality) [18,19]. Two researchers independently reviewed the interview materials, summarized and extracted meaningful statements. Disagreements about the contents were discussed and resolved with a Professor (LL). Interrater reliability between two graduate students was calculated with Cohen’s kappa coefficient (κ).

## 3. Results

### 3.1. Quantitative Study

In the quantitative study of medical students involved in epidemic prevention and control, the average age was 21.34 (SD 2.0) years, and there were more female students (52.4%) than male students (47.6%). Approximately 70% of the participants were lower-grade students (Table 1).

**Figure 1:**
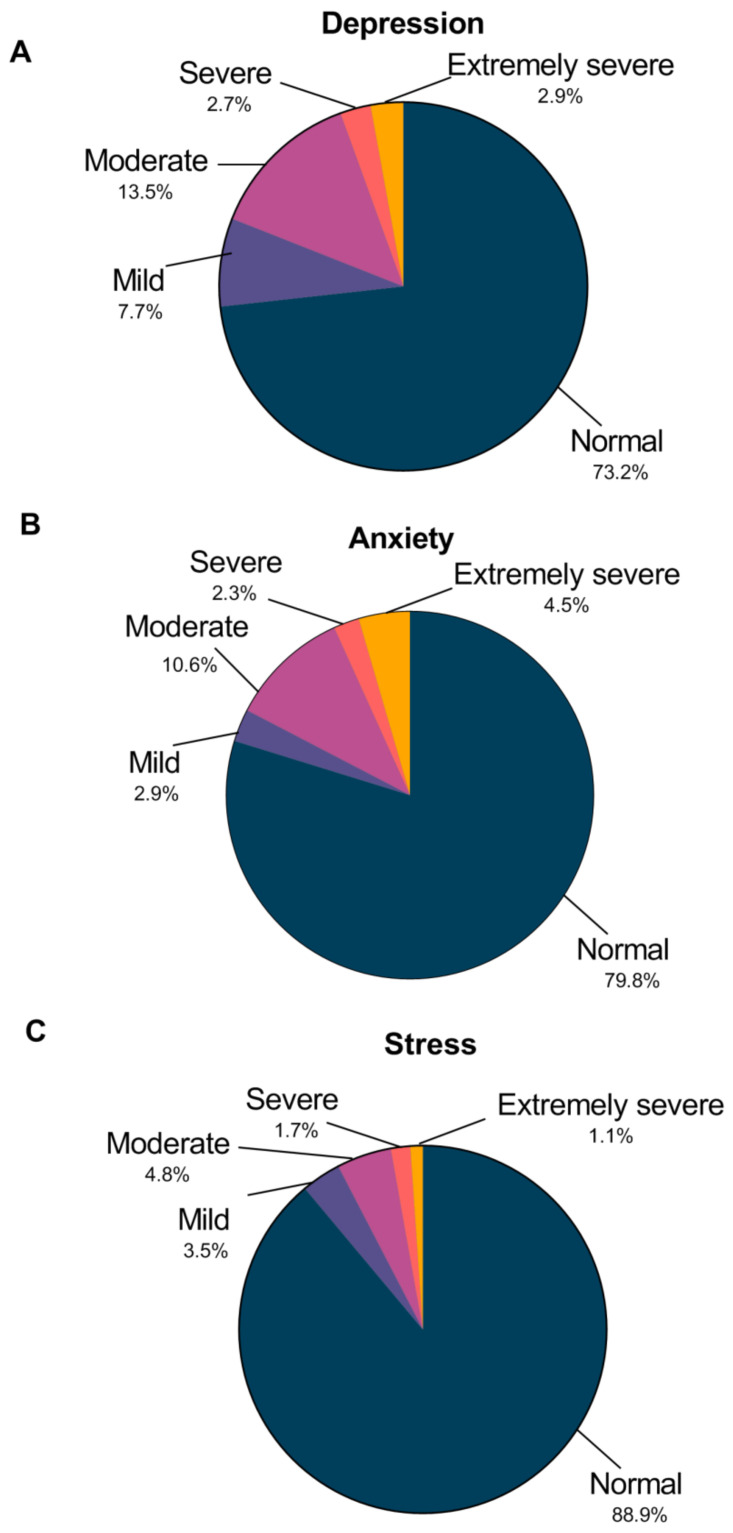
The proportion of participants whose answers on the DASS-21 indicated a normal, mild, moderate, severe or extremely severe amount of depression (**A**), anxiety (**B**), and stress (**C**).

As shown in Figure 1, the scoring of the DASS-21 ranks each participant’s depression, anxiety, and stress levels, classifying each area as either “normal”, “mild”, “moderate”, “severe”, or “extremely severe”. Our study found that the detection of depression, anxiety and stress of medical student volunteers were 26.8%, 20.2% and 11.1%, respectively. In total, 5.6% of medical students reported severe-to-extremely severe depression, 6.8% indicated severe-to-extremely severe anxiety, and 2.8% reported symptoms of severe-to-extremely severe levels of stress.

### 3.2. Qualitative Study

The interrater reliability between the coding teams was κ = 0.894, indicating almost perfect agreement [20].

In our qualitative research, we enrolled four males and five females between the age of 18 and 29. There were four clinical medicine students, two students majoring in epidemiology and health statistics and three public health students. Table 2 outlines the baseline characteristics of the participants. A content analysis of data obtained through the qualitative study regarding the psychological burden and experiences of medical student volunteers during the COVID-19 pandemic led to the identification of two to five themes for each category of outcomes. Exemplar quotes for each theme were displayed in Table 3 and Table 4.

#### 3.2.1. Psychological Burden

*Exposed to suspected/confirmed cases*: A small number of study subjects (*n* = 2) experienced a significant amount of negative emotions in the early stage, especially when in contact with patients for the first time. A participant specifically mentioned that the COVID-19 outbreak made them feel close to the disease. It was the first time they experienced such a large infectious disease. Many participants (*n* = 6) remained optimistic during the COVID-19 epidemic, believing that as long as personal protection was well done, there was no need to worry too much.

*During the volunteer service period*: One participant expressed her concern for family members whose occupations, such as primary care workers and health care workers, increase their risk of exposure to COVID-19. Although most medical student volunteers had negative emotions, such as fear, anxiety, and worry, for the first time, their negative emotions would gradually faded away. After a week, some of them showed more positive emotions. Only one or two interviewees said they were in a state of anxiety throughout the entire epidemic, which they described as being caused by academic troubles and a lack of protective equipment.

*Worries related to infection*: Most of the members indicated they were not worried about COVID-19 infection during the epidemic prevention and control period, believing that their environment and positions were safe. Some participants said they were more worried about transmission to their families than themselves. Only two volunteers, working in areas with serious outbreaks, said they were slightly concerned about their own health due to repeated exposure to suspected/confirmed cases.

*Source of confidence*: All participants expressed confidence that China would overcome the epidemic. This confidence came from the reduction in mortality, the support of the provinces, the rapid development of vaccines and the encouragement of the leaders. Some interviewees said that, during the epidemic, the government’s announcements regarding progress in vaccine development had a positive effect on their psychology and strengthened in relation to the idea of beating COVID-19.

#### 3.2.2. Experience

*Reasons for signing up as a volunteer*: One interviewee took the initiative to participate in the prevention and control of the epidemic because of the occupation of her parents. Parents who were in the medical profession encouraged their children of medical students to actively participate in medical activities within the limits of their ability. Approximately half of the participants (4/9) said that rather than remaining at home in isolation, they were more willing to participate in social activities and used their knowledge to serve the community. Some volunteers (*n* = 4) said that they participated in the prevention and control of the epidemic due to the fact that they felt a responsibility as medical students. Most of the participants (*n* = 7) chose to be a volunteer with the encouragement of their families or teachers. There was also volunteers who chose to participate because his friends had also taken part in volunteering activities.

*Participating again*: All students expressed their willingness to use their knowledge to participate in volunteer activities again, if necessary. On the one hand, as medical students, some believed that they should use what they had learned to reduce the burden on medical workers. On the other hand, some also thought that they would be more skillful in their future profession because they had some experience in epidemic prevention and control. Another volunteer mentioned that she was willing to participate, but would consider where she would participate more carefully next time, e.g., if she needed to support a hospital in an infected area, she would assess whether she had the ability to do so and she wanted to participant after she had the ability to do so.

*Impact on career*: The majority of participants (*n* = 6) said that volunteering had no impact on their careers and would not change their career direction or result in them not pursuing a professional-related work as a result of the epidemic. A small number of medical student volunteers said they would be more determined to engage in medical-related careers. One medical student hoped to make a transition to academic research.

## 4. Discussion

To the best of our knowledge, this was the first study to use a combination of quantitative and qualitative methods to examine the psychological influences and experiences of COVID-19 among medical student volunteers in China. The present study found that the highest levels of depression, anxiety and stress among medical student volunteers were 5.6%, 6.8% and 2.8%, respectively. Our findings were lower than some studies, such as a study by Iqbal S. et al. on medical undergraduate students [21] and Zhou S.J.’s on Chinese adolescents during the COVID-19 outbreak [22]. This discrepancy may be due to the different timings of the surveys and the fact that one of the survey was carried out in a different survey country.

The findings of this study showed a low rate of psychological distress among medical student volunteers involved in outbreak prevention and control, that is, most were able to maintain a positive mindset. Medical students have a higher perceived sufficiency of information on the prognosis and transmission of COVID-19. During the epidemic prevention and control period, medical students were able to reduce their psychological distress to some extent by using their knowledge to promote prevention and control to those around them, while ensuring that they also remained protected [23,24]. Moreover, when the epidemic was slightly alleviated, the Chinese government advised that medical students acted as contributors in certain low-risk areas, to alleviate the burden on medical personnel. In our qualitative analyses of interviewed medical students, some reported bad moods were alleviated after they went out to participate in work. The low incidence of psychological burden among students might also be attributed to online teaching methods. Some studies have found that many reasons for high anxiety level at baseline were related to academic issues, and online learning might have eased the overburdened academic courses [23]. Our quantitative survey was conducted in April 2020, when students began using online teaching methods, which may have contributed somewhat to the improved mental health status of the medical students surveyed in this study compared to other similar surveys.

Our qualitative research explored deeply and holistically understood the meaning of COVID-19 volunteer activities for medical students using the framework analysis method. After analyzing the interview data from medical students who signed up as COVID-19 volunteers, the psychological and experiential aspects of their encounters were identified.

COVID-19 is a new and highly infectious disease that has spread rapidly across the world [25]. During the COVID-19 outbreak period, with the increasing number of cases and the shortage of medical personnel, Stokes [26] raised the issue of whether senior medical students would be involved in emergency relief efforts. This measure might not only improve the medical students’ ability to deal with sudden infectious diseases and have more experience in relevant medical work in the future, but might also alleviate the shortage of hospital personnel. However, due to their imperfect medical knowledge and the lack of clinical experience, medical students tended to have negative emotions when they participate in medical care-related work. Our results indicated that medical student volunteers suffered from a psychological burden when they engaged in voluntary activities, but that their overall mental health status was good. We found that medical student volunteer’s negative emotions were not present at the time of exposure to suspected/confirmed cases, but were more pronounced before accepting the mission. Most volunteers said that, after entering the workplace to assist the related staff, they felt calm and relaxed. This finding aligned with the results reported in many studies [27,28], that is, negative emotions in the face of an epidemic would be more obvious until the epidemic prevention efforts were underway, at which point they gradually disappeared, with positive emotions emerging. Therefore, early mental health interventions are particularly important for medical student volunteers during a public health emergency [29]. Many studies have provided an overview of important considerations, such as the fact that inadequate personal protective equipment could cause psychological trauma for medical professionals [30]. A survey of the psychological state of medical workers in Spain showed that [31] 85.4% stated that the lack of personal protective equipment led to increase stress and anxiety. Medical students who enrolled in voluntary activities in this study said that insufficient personal protective measures, such as insisting on wearing masks during work and keeping distance from others, had a comforting effect on their psychology and psychological distress would be reduced accordingly.

Throughout the volunteer’s activities during the epidemic, participants were most worried about infecting their family members rather than contracting COVID-19 themselves. Volunteers were not only worried that their volunteer activities would result in them transmitting the disease to their loved ones, but also that their family members, whom were working on the front line, might be infected by a patient. Other previous similar studies [31,32] have also reported that alongside concerns for their physical health, health-care workers were also concerned about transmitting an infection to their families.

Our study also found that, during volunteering activities, students were concerned about academic postponement due to the outbreak. As the student’s participation in volunteer activities was concentrated between February and April 2020, a period when schools were in lockdown and information about the start of school was not communicated, it was also psychologically important for students to take personal precautions, to maintain stable and close relationships with their families, and to return to normal life as soon as possible.

In the case of the COVID-19 outbreak, the reduction in mortality, the support of the provinces, the rapid development of the vaccines and the encouragement of the leaders were all factors that made medical student volunteers felt confident that the measures taken against the epidemic would be successful and that zero cases could be achieved. Firstly, after the outbreak of the COVID-19 in 2020, the Chinese government quickly responded, imposing a restricted blockade policy on Wuhan, a “one province to one city” policy—a one-to-one support relationship to mobilize medical teams from other provinces to support the epidemic area in Hubei. These strategies not only eased the burden on medical personnel in the infected areas, but also effectively increased the recovery rate and contained the spread of the epidemic. Secondly, there were no treatments for this disease, and various countries were stepping up the research and development of the vaccine in the early stages of the outbreak, while on 22 May the results of human clinical trials of a COVID-19 vaccine by Chinese academician Chen was published [33]. This successful development of the vaccine therefore boosted the confidence of volunteers against the epidemic. Thirdly, the encouragement of the leaders and the positive mindsets of the colleagues around them had a great impact on the volunteers. Studies of health-care workers by Sun et al. [27] and by Khalid [34] also indicated that a positive attitude from team colleagues and peers could ease the psychological burden during attempts to fight against epidemics. To reduce the negative psychological impact of the volunteering activities on the students, positive emotions and mutual encouragement from the team were important.

The participants were motivated by their duties as medical students, the encouragement of their families/teachers, and the fact that they had been isolated at home for too long. They felt pride as volunteers in providing support and fulfilling their responsibilities during a time of national crisis. Additionally, participant’s statements confirming that they would volunteer again were consistent with the study results of Kim among nurses [28]. The vast majority indicated that involvement in pandemic preparedness and control would not have an impact on their career, that is, it would not change their commitment to pursue a role in the medical profession. Due to the drastic surge of COVID-19 patients around the world, many countries were considering or had graduated advanced health-care students early so that they could join the workforce [35]. The experience of the current pandemic has shown the willingness of the vast majority of medical students to adopt health-care assistant roles, not only because of their status as medical students, but also because this behavior was encouraged thank to the support of those around them. Medical students were willing to use their medical expertise to contribute what they could to response efforts and to increase their experience in emergency situations when the country needed them.

This study has the following limitations. First, this study utilized in-depth interviews to collect data. Participant’s responses may be influenced by the interviewer’s interview technique, resulting in results that might appear to be guided responses. However, the researchers conducting the interviews were trained in appropriate interview techniques, and we used semi-structured and open-ended questions to mitigate this limitation. Second, the qualitative research data were collected 6 months after the medical student volunteers had left the workforce. This time delay might have caused some recall bias. We attempted to recruit students who had worked for a longer of time in volunteer activities related to epidemic prevention in order to increase the validity of the data. Third, the result of the quantitative analyses did not include more factors potentially influencing medical student psychological burden.

## 5. Conclusions

This study provides a comprehensive insight into the psychology of medical student volunteers and their experiences during COVID-19 through qualitative and quantitative methods. The overall mentality of Chinese medical student volunteers was in good—most of them showed negative emotions in the early stages of participating in the epidemic prevention and control work, and then gradually declined, while simultaneously positive emotions appeared. The vast majority of medical students were willing to volunteer as medical assistants when their country needed them due to their sense of responsibility as medical student. This study on the psychological and experiential aspects were directly derived from Chinese medical student volunteers and may have a significant impact on the response to future public health emergencies in similar settings.

## Figures and Tables

**Table 1:** Baseline characteristics of participants (*n* = 1041).

Variables	Volunteer
Gender, *n* (%)	
Male	496 (47.6%)
Female	545 (52.4%)
Age (years), mean (SD)	21.34 (2.0)
Academic year, *n* (%)	
Lower Grade	684 (65.7%)
Higher Grade	292 (28.0%)
Postgraduate stage	65 (6.2%)

**Table 2:** The baseline characteristics of the participants.

Participant Number	Age (Years)	Sex	Education	Major	Tasks Performed during COVID-19	Work Setting
P1	24	Male	Graduate status	Clinical medicine	Took the temperature of others at the road junctions; Disseminated the knowledge of epidemic prevention and control	The districts of temperature monitoring at sub-district Office
P2	26	Female	Graduate status	Epidemiology and health statistics	Collated the data of suspected cases; Disinfected and sterilized the epidemic area	Centers for disease control and prevention
P3	26	Female	Graduate status	Public health	Took the temperature of others at the road junctions; Checked in register visitors	Neighbourhood
P4	27	Female	Graduate status	Public health	Collected nucleic acids	Centers for disease control and prevention
P5	22	Male	Undergraduate status	Clinical medicine	Collected nucleic acids; Assist medical staff to examine patients	Isolation rooms
P6	29	Male	Graduate status	Epidemiology and health statistics	Conducted an epidemiological survey; Collated the data of suspected cases	Centers for disease control and prevention
P7	20	Female	Undergraduate status	Clinical medicine	Took the temperature of others at the road junctions;	The districts of temperature monitoring at the exit of the highway.
P8	27	Male	Graduate status	Clinical medicine	Conducted an epidemiological survey; Disinfected and sterilized the epidemic area; Disseminated the knowledge of epidemic prevention and control	Centers for disease control and prevention
P9	18	Female	Undergraduate status	Stomatology	Disseminated the knowledge of epidemic prevention and control; Collected health information from residents	Community

**Table 3:** Psychological impact by medical student volunteers.

Categories	Sub-Categories	Example Citation
**What’s the mentality when exposed to suspected/confirmed cases?**		
	Significant amount of negative emotions in the early stage	P5: “I was nervous when I first came into contact with a confirmed case.” P9: “I was a little scared at first. It was at the beginning of the outbreak.”
	Have a sense of crisis	P1: “Everything becomes very real. We had been talking about the epidemic before, but we didn’t know where it was until a case appeared in our community.”
	Keep optimistic attitude	P8: “Because at that time, I also keep adequate protective equipment, such as masks, medical-use insulation garment, etc., so I didn’t be too much fear.”
**What is the state of emotion during the volunteer service period?**		
	Fear of infecting the families	P1: “I am worried that I will infect my parents” P9: “My parents are medical workers, and I am worried that they will be infected as a result of contact with patients.”
	Significant amount of negative emotions in the early stage.	P2: “Because I don’t know what kind of situation I will face, so I will be nervous when I went to CDC on the first day.” P5: “I was a little depressed at that time, … Because of the death of a patient in the ward, I would dream of the patient asking for help during that time.”
	Anxious during the whole epidemic.	P4: “I follow the news every day. I was nervous at that time and worried about can not go back to university.” P7: “I didn’t take it seriously at first, but then I was very anxious when I couldn’t get the mask.”
**Whether be worried about being infected?**		
	Not worried about infection	P3: “I don’t think so. I felt that my environment was still relatively safe at that time.” P6: “Because I majored in medicine, I don’t think I should get too close to patients. Pay attention to myself. It should not be a big problem.”
	Fear of infecting the family	P1: “Before I went home, I thought whether the contact I had with so many people today would have some impact on my family.”
	A little fear of infection throughout the period	P4: “Once someone clears his throat, I got inexplicably nervous.” P8: “Just a little worried.”
**What was the source of the confidence in defeating COVID-19 at that time?**		
	The reducing mortality	P5: “Because we work here, we can also find that we receive fewer and fewer patients every day.”
	The support of the provinces	P9: “Because I am from Hubei, at that time, many provinces sent supplies and medical personnel to support us.”
	The hopes on the vaccine	P7: “Until later, I heard that COVID-19 vaccine had been developed, and then slowly began to have confidence.”
	The encouragement of the leadership	P8: “At the end of each morning meeting, the director of CDC would say some inspiring words, …and every time I heard about this, I thought that what we did must be able to overcome the epidemic in the end.”

**Table 4:** Experience for medical student volunteers.

Categories	Sub-Categories	Example Citation
**Why did you sign up for volunteering?**		
	The medical professionals’ identity of parents	P9: “My parents, both health care workers, took part in the fight against the epidemic. They worked very hard every day, and sometimes they were too busy to go home. I want to lighten their burden.”
	Home isolation for too long	P5: “At that time, I thought it would be better to make a contribution instead of staying at home all the time.”
	Strong responsibility as a medical students	P4: “First of all, I majored in this major public health.” P6: “Because of the responsibility of my position, I am a student majoring in epidemiology and health statistics.”
	Encouragement from family/teachers	P2: “Our teacher suggested that we could use what we have learned to participate in voluntary activities.” P4: “When I signed up for the volunteer activity, my father encouraged me, saying that it was all right.”
	Peer pressure	P1: “My classmates volunteered in their hometown.”
**If the epidemic breaks out again, will you still take part in the fight against the epidemic?**		
	I will participate but consider whether I have the ability	P7: “If the hospital needs manpower in the next outbreak, I think I will consider whether I have professional skills and a reserve of knowledge. If I don’t have enough knowledge to go to the hospital, I will be more worried about my situation and bring inconvenience to the medical staff.”
	I will participate due to the duties of medical students.	P2: “As medical students, we should have contribute to the COVID-19. Therefore, if there is any need, I believe I am duty-bound.”
	I will attend because of my experience in fighting the epidemic.	P4: “I have experience in CDC, and I will be willing to do it when I am needed.”
**Whether it has an impact on career or not?**		
	No impact on career	P5: “I won’t switch to respiratory medicine because of this. I still prefer cardiology.”
	Be more determined to engage in the medical profession	P4: “I think this epidemic makes me feel that I have a positive role, and that I am more determined to engage in my professional work.”
	Shift from clinical practice to scientific research.	P1: “I will make use of the scientific research knowledge I have learned now to provide some help as much as I can.”

## Data Availability

The dataset used and/or analyzed during the current study are available from the corresponding author on reasonable request.
